# The Addition of Dydrogesterone after Frozen Embryo Transfer in Hormonal Substituted Cycles with Low Progesterone Levels

**DOI:** 10.1055/s-0042-1751058

**Published:** 2022-11-29

**Authors:** Jose Metello, Claudia Tomas, Pedro Ferreira, Samuel Santos-Ribeiro

**Affiliations:** 1 Hospital Garcia de Orta, Cirma, Almada, Portugal.; 2 Ginemed, Ginemed-Lisboa, Lisboa, Portugal.; 3 Instituto Valenciano de Infertilidade (IVI-RMA) Lisboa, Lisboa, Portugal.

**Keywords:** dydrogesterone, frozen embryo transfer, artificial cycle, assisted reproductive technology outcomes, luteal phase, didrogesterona, transferência de embriões congelados, ciclo artificial, sucesso em procriação medicamente assistida, fase lútea

## Abstract

**Objective** To determine whether a rescue strategy using dydrogesterone (DYD) could improve the outcomes of frozen embryo transfer cycles (FET) with low progesterone (P4) levels on the day of a blastocyst transfer.

**Methods** Retrospective cohort study including FET cycles performed between July 2019 and October 2020 following an artificial endometrial preparation cycle using estradiol valerate and micronized vaginal P4 (400 mg twice daily). Whenever the serum P4 value was below 10 ng/mL on the morning of the planned transfer, DYD 10 mg three times a day was added as supplementation. The primary endpoint was ongoing pregnancy beyond 10 weeks. The sample was subdivided into two groups according to serum P4 on the day of FET: low (< 10 ng/mL, with DYD supplementation) or normal (above 10 ng/mL). We performed linear or logistic generalized estimating equations (GEE), as appropriate.

**Results** We analyzed 304 FET cycles from 241 couples, 11.8% (n = 36) of which had serum P4 below 10 ng/mL on the FET day. Baseline clinical data of patients was comparable between the study groups.

Overall, 191 cycles (62.8%) had a biochemical pregnancy, of which 131 (44,1%) were ongoing pregnancies, with a 29,8% miscarriage rate. We found no statistically significant differences in the hCG positive (63 vs 64%) or ongoing pregnancy rates (50 vs 43,3%) between those FETs with low or normal serum P4 values, even after multivariable logistic regression modelling.

**Conclusion** Our results indicate that DYD 10 mg three times a day administered in women who perform FET with P4 serum levels < 10 ng/mL, allows this group to have pregnancy rates beyond 12 weeks at least as good as those with serum levels above 10 ng/mL.

## Resumo

**Objetivo** Determinar se uma estratégia de resgate usando didrogesterona (DYD) pode melhorar os resultados dos ciclos de transferência de embriões congelados (TEC) com baixos níveis de progesterona (P4) no dia de uma transferência de blastocisto.

**Métodos** Estudo de coorte retrospectivo que incluiu ciclos TEC realizados entre julho de 2019 e outubro de 2020 após um ciclo de preparação endometrial artificial usando valerato de estradiol e P4 vaginal micronizado (400 mg duas vezes ao dia). Sempre que o valor de P4 sérico estava abaixo de 10 ng/mL na manhã da transferência planejada, adicionou-se 10 mg de DYD tri-diário como suplementação. O desfecho primário foi gravidez evolutiva após 10 semanas. A amostra foi subdividida em dois grupos de acordo com o P4 sérico no dia da TEC: baixo (< 10 ng/mL, com suplementação de DYD) ou normal (acima de 10 ng/mL). Realizamos equações de estimativa generalizada linear ou logística (GEE), conforme apropriado.

**Resultados** Analisaram-se 304 ciclos de FET de 241 casais, dos quais 11,8% (n = 36) tinham valores de P4 sérico abaixo de 10 ng/mL no dia da TEC. Os dados clínicos e demográficos dos pacientes eram comparáveis entre os grupos.

Globalmente, 191 ciclos (62,8%) tiveram uma gravidez bioquímica, dos quais 131 (44,1%) foram gestações em curso, com uma taxa de aborto espontâneo de 29,8%. Não encontramos diferenças estatisticamente significativas na taxa de gravidez bioquímica (63 vs. 64%) ou nas taxas de gravidez evolutiva (50 vs. 43,3%) entre TEC com valores séricos de P4 baixos ou normais, mesmo após modelação com regressão logística multivariável.

**Conclusão** Nossos resultados indicam que a suplementação com DYD 10 mg três vezes ao dia em mulheres com níveis séricos de P4 abaixo de 10 ng/mL em ciclos de TEC substituídos parecem conseguir resultados pelo menos tão bons como nos ciclos com valores superiores para taxas de gravidez em curso além de 12 semanas.

## Introduction

Frozen embryo transfers (FETs) have increased considerably over the past decade. 1 Endometrial preparation in FETs can be achieved in natural, modified, or artificial cycles, but up to now there is no recommended evidence to support one protocol over another. 2 3 4 Nonetheless, some evidence is building on the benefits of the corpus luteum, especially its influence on neonates' size, and its ability to reduce hypertensive disorders in pregnancy. 5 6 7

In hormone-substituted cycles for FET, the administration of exogenous progesterone (P4) after estrogen priming plays a fundamental role in the secretory transformation of the endometrium, given the absence of any endogenous production of P4 by the corpus luteum, 2 or placenta, until at least the 7 ^th^ to 9 ^th^ week of gestation. 8 9

In FET cycles, estradiol is often given in patches, oral, or vaginal pills, while exogenous P4 may be administered orally, vaginally, subcutaneously, or intramuscularly. The vaginal and intramuscularly administration have both been extensively studied, with neither showing superiority over the other in terms of pregnancy outcomes. 10 Although the vaginal route is often preferred, 11 12 many have advocated the need for serum P4 monitoring during these FET cycles given that the absorption and metabolization in each patient is very variable. 13 14 15 More recently, the oral route has also been evaluated as a potentially noninferior alternative, at least when used in fresh embryo transfers. 12 16 17

During the last years, several retrospective and prospective studies have demonstrated a relationship between low serum P4 levels on the day of embryo transfer and a reduction in ongoing and delivery rates. 13 14 18 19 20 21 22 23 In such cases, researchers have advocated the use of enhanced P4 “rescue” strategies, although evidence of the benefit of such an approach is currently lacking.

Dydrogesterone (DYD) is an orally administered synthetic molecule. Despite having a bioavailability lower than 10% following first hepatic passage, 15 it is still significantly more active than orally administered micronized P4, given its high selectivity for the P4 receptor, 24 25 allowing it to be used in much lower doses with lesser side effects. 12 26 The active metabolite of DYD is 20-alpha-dihydrodydrogesterone, which has a half-life of up to 17 hours, with no additional agonist or antagonist activity in other receptors. That said, the main drawback of this drug is the difficulty of monitoring serum 20-alpha-dihydrodydrogesterone levels. 8

We sought to determine whether a rescue strategy with DYD starting immediately after a blastocyst transfer could improve the outcome of FET cycles with low P4 values on the day of a blastocyst transfer.

## Methods

This was a retrospective study conducted at the Centro de Infertilidade e Reprodução Medicamente Assistida (CIRMA) between July 2019 and October 2020. We included only FET cycles in women aged between 18 and 40 years who transferred 1 or 2 frozen blastocysts with an expansion degree equal to or greater than 2 and with a grade 1 or 2 internal cell mass and trophectoderm (Istanbul Consensus, 2011) 27 in a hormone-substituted cycle.

Patients with an endometrial thickness below 6 mm prior to P4 administration, untreated endocavitary disease, uncorrected Müllerian anomaly, or those who were demonstrated to have serum P4 values below 3 ng/mL on the day of embryo transfer (n = 2) were excluded from analysis.

The study was approved by the local Institutional Review Board.

On the second day of a spontaneous or post-pill menstrual cycle, patients started estradiol (Zumenon, Bayer Portugal, SA., Carnaxide, Portugal) at a dose of 2 mg every 12 hours vaginally, with the first transvaginal ultrasound control being performed 12 to 20 days later. If the endometrial thickness was over 6 mm, with resting ovaries, patients started vaginal administration of P4 (Progeffik, Laboratórios Effik, Algés, Portugal) at a dose of 400 mg every 12 hours starting on the following morning. Whenever necessary, luteinizing hormone (LH) and P4 serum assessments were performed in order to exclude the occurrence of spontaneous ovulation prior to starting exogenous P4 administration.

Another serum P4 assay was performed on the morning of the transfer after the 11 ^th^ vaginal P4 administration. Whenever the P4 value was below 10 ng/mL, 10 mg of DYD (Duphaston, BGP products LTD., Lisboa, Portugal) three times a day was started and maintained until at least 8 weeks of pregnancy. The cut-off used for DYD administration was set according to a previous evaluation of our own population, which corresponds to 11.5% of the pool of the cycles. 28 In this cohort women with lower progesterone levels had a trend towards lower delivery rates (26 vs. 39%; *p* > 0.05).

A maximum of two good quality blastocysts (ALPHA group) were warmed according to the following protocol: the Cryotop straw (Kitazato Corp., Shizuoka, Japan) was removed from liquid nitrogen and immediately submerged in 300µl thawing solution (Kitazato Corp., Shizuoka, Japan), previously heated to 37˚C. After 1 minute, the embryos were placed in a 60µl drop of diluent solution (Kitazato Corp., Shizuoka, Japan) for 3 minutes at room temperature. Finally, the embryos were placed in a 60µl drop of washing solution (Kitazato Corp., Shizuoka, Japan) for 5 minutes at room temperature, and then washed for 1 minute in another drop of 60µl washing solution at room temperature. They were then placed into 30µl drops of Sequential Blast medium (CooperSurgical Fertility Solutions, Måløv, Denmark) and covered with Liquid Paraffin (CooperSurgical Fertility Solutions, Måløv, Denmark), where they remained for at least 2 hours prior to transfer.

Embryo transfers were routinely performed under ultrasound guidance using either Cook or Wallace embryo catheters introduced until passing the middle of the endometrial cavity, where the embryos were later deposited. The β-HCG test was performed 9 to 12 days after the transfer and, in those who conceived, estradiol and DYD was maintained until at least until the 8 ^th^ week of pregnancy and vaginal progesterone until the 10 ^th^ week.

The P4 hormone assays were performed using the electrochemiluminescence (ECLIA) and the Cobas 8000 Roche Diagnostics (Hoffmann–La Roche AG., Switzerland) equipment in the morning (between 9–11 am ).

A hCG positive pregnancy was diagnosed in all cycles with a serum β-HCG value > 10 IU/L. Meanwhile, an ongoing pregnancy was defined as the presence of at least one embryo with heart beating detected after the 12 ^th^ week of pregnancy. Miscarriage was defined as hCG positive pregnancy that did not go beyond the 12 ^th^ week.

Cycles with progesterone values below 10 ng/mL were compared with those above. The following variables were subject to comparative statistical analysis between the study groups: female and male age on the day of the oocyte retrieval, female age on the FET day, infertility duration, female weight, female body mass index (BMI), female and male smoking habits, female and male ethnicity, serum anti-Müllerian hormone (AMH) values, antral follicle count (AFC), total dose of gonadotropins used in the IVF/ICSI cycle, number of oocytes collected and used, number of embryos obtained, blastocyst development day (D5 or D6), number of transferred embryos, FET rank, endometrial thickness prior to FET, and serum P4 value on the day of FET.

Using the Statistical Package Social Sciences (SPSS, IBM Corp., Armonk, NY, USA) software version 22.0, we performed linear or logistic generalized estimating equations (GEE), as appropriate, in order to account for the inclusion of more than one FET cycle performed by the same patient. 29 A *p* -value below < 0.05 was considered statistically significant.

Finally, for the outcome ongoing pregnancy, we also performed multivariable GEE in order to account for potential confounding factors, with all variables in which the univariable model presented a *p* < 0.15 being included in the final multivariable model.

## Results

Overall, a total of 304 cycles from 241 couples were included. There was a total of 191 (62.8%) pregnancies, of which 131 (44.1%) were ongoing pregnancies, with a 29.8% miscarriage rate. Among these, 36 (11.5%) had P4 levels below 10 ng/mL on the day of FET and, hence, were administered DYD 10 mg three times a day in addition to vaginal micronized P4. These were compared with cycles with progesterone ≥ 10 ng/mL.

### Comparison of the Cycles with and without DYD Supplementation

Table 1 shows the baseline clinical data of patients according to serum progesterone levels on the day of embryo transfer. Overall, women with serum P4 levels < 10 ng/mL weighed more. There were no other statistically significant differences between the study groups amongst the other variables evaluated.

**Table 1.** Baseline characteristics of both groups according to the progesterone value on the day of embryo transfer ≥ or < than 10 ng/mL

	Progesterone ≥ 10 ng/mL (n = 268)	Progesterone < 10 ng/mL (n = 36)	*p* -value
Female age at pickup (years), mean ± SD	34.1 ± 3.6	33.8 ± 3.5	0.64
Female age at FET (years), mean ± SD	35.0 ± 3.6	34.4 ± 3.6	0.40
Infertility duration (months), mean ± SD	60.1 ± 30.2 (n = 265)*	65.5 ± 34.0	0.40
Endometrium thickness (mm), mean ± SD	9.7 ± 1.8 (n = 253)*	9.8 ± 1.7 (n = 30)*	0.79
Female weight (kg), mean ± SD	63.2 ± 12.7	69.5 ± 14.4	0.03
Female BMI (kg/m ^2^ ), mean ± SD	24.1 ± 4.7	25.8 ± 5.5	0.10
Male age at pickup (years), mean ± SD	36.0 ± 4.5 (n = 265)*	37.5 ± 7.4	0.30
AMH (ng/mL), mean ± SD	3.7 ± 3.7 (n = 264)*	4.3 ± 4.2 (n = 35)*	0.42
AFC, mean ± SD	18.7 ± 9.8 (n = 263)*	20.2 ± 11.8	0.49
Gonadotrophins dose (UI), mean ± SD	2606.3 ± 814.5	2626.9 ± 793.5	0.88
Oocytes collected, mean ± SD	15.5 ± 8.2	15.8 ± 7.0	0.82
Oocytes used, mean ± SD	14.3 ± 7.4	14.8 ± 6.6	0.66
2PN, mean ± SD	9.8 ± 5.7	8.9 ± 4.7	0.30
Female smoking			0.06
Never	144 (53.7%)	26 (72.2%)	
Current/past	124 (46.3%)	10 (27.8%)	
Male smoking			0.69
Never	108 (40.8%)	16 (44.4%)	
Current/past	157 (59.2%)	20 (55.6%)	
Female race			0.54
Non-Caucasian	32 (11.9%)	3 (8.3%)	
Caucasian	236 (88.1%)	33 (91.7%)	
Male race			
Non-Caucasian	29 (10.9%)	3 (8.3%)	0.65
Caucasian	236 (89.1%)	33 (91.7%)	
FET rank			0.71
1	180 (67.2%)	25 (69.4%)	
2	67 (25.0%)	9 (25.0%)	
3	15 (5.6%)	2 (5.6%)	
4	5 (1.9%)	0 (0.0%)	
5	1 (0.4%)	0 (0.0%)	
Transfer day			
Day 5	216 (80.6%)	30 (83.3%)	0.70
Day 6	52 (19.4%)	6 (16.7%)	
Number of embryos transferred			0.81
1	213 (79.5%)	28 (77.8%)	
2	55 (20.5%)	8 (22.2%)	

**Abbreviations:** 2PN, two-pronuclear zygote; AFC, antral follicle count; AMH, anti-Müllerian hormone; BMI, body mass index; FET, frozen embryo transfer; SD, standard deviation. **Notes** : * Excluded cases with missing values.

### Clinical Outcomes According to the Progesterone Values and DYD administration

**Fig. 1.**
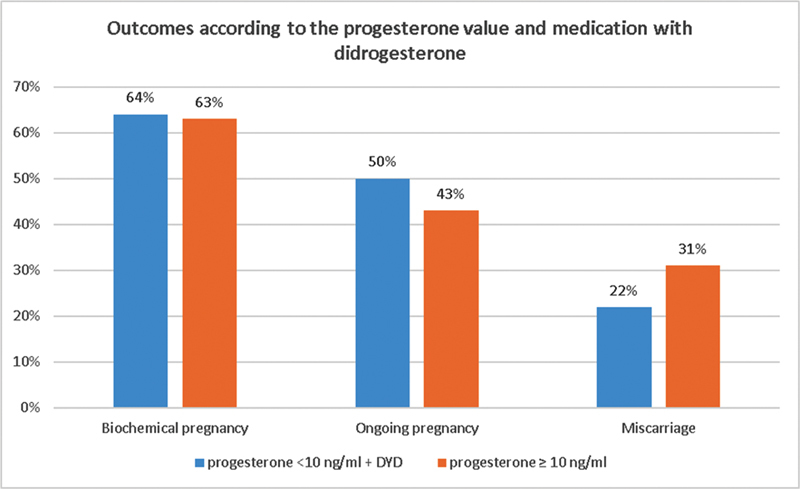
Outcomes according to the groups: progesterone < 10 ng/mL with addition of DYD three times a day versus progesterone > 10 ng/mL.

Figure 1 shows the outcomes according to the groups: progesterone < 10 ng/mL with DYD addition versus progesterone > 10 ng/mL. No statistically significant differences were found concerning biochemical pregnancy (64 vs 63%; *p* = 0.889), ongoing pregnancy (50 vs 43%; *p* = 0.446), and miscarriage (22 vs 31%; *p* = 0.365) rates.

### Clinical Outcomes According to Progesterone Distribution

**Fig. 2.**
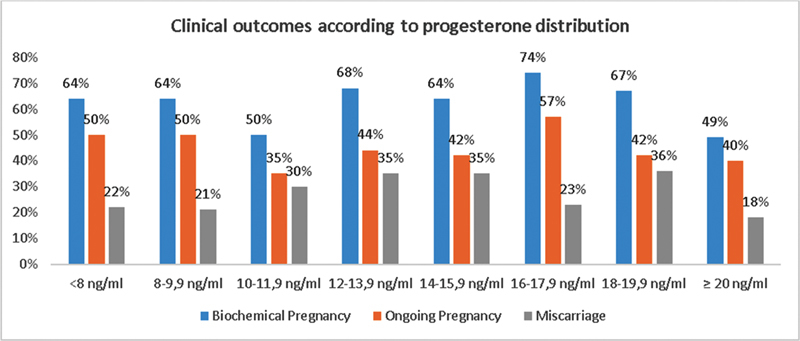
Outcomes according to the percentile of progesterone distribution.

The P4 serum concentration on the day of the embryo transfer varied between 4.4 and 32.3 ng/mL, with an average of 14.7 ng/mL. Figure 2 shows a sensitivity analysis with the distribution and outcomes according to different P4 intervals (presented in regular intervals of 2 ng/mL).

Taking into account the sensitivity analysis, we decided to subdivide the group with P4 ≥ 10 ng/mL into an upper and lower group. For this, we used the cut-off on 20% (12.2 ng/mL) of the sample ( Table 2 ). Cycles with a lower level of serum progesterone had significantly lower hCG positive pregnancy rates. Finally, we also performed stepwise logistic GEE regression using all the variables with a *p* -value below 0.15 in the univariable analysis for the outcomes ongoing pregnancy rate: AMH, transfer day, number of embryos transferred, plus female age at pickup, and progesterone value below or above 10 ng/mL ( Table 3 ). In this final model, only the number of transferred embryos was significantly associated with ongoing pregnancy rate. More importantly, we also found that the use of DYD and a serum progesterone level below 10 ng/mL on the day of embryos transfer was not associated with the outcome in the adjusted model.

**Table 2.** Sub-group evaluation

	P4 10–12.2 ng/mL (n = 53)	P4 12.3–32.3 ng/mL (n = 215)	*p* -value
βHCG +	49.1%	66.0%	0.026
Ongoing pregnancy	34.0%	45.6%	0.130
Miscarriage	30.8%	31.0%	0.181

**Notes:** The 20% cycles with lower serum P4 values that did not have the addition of DYD.

**Table 3.** Adjusted odds ratio for clinical pregnancy rate

	Univariable			Multivariable		
	OR	95% CI	*p* -value	OR	95% CI	*p-* value
Female age at pickup	0.952	(0.895–1.014)	0.125	0.948	(0.886–1.014)	0.121
AMH	1.054	(0.991–1.120)	0.092	1.051	(0.986–1.121)	0.125
Transfer day (D5 vs. D6)	1.776	(0.978–3.22)	0.059	1.635	(0.879–3.041)	0.120
Number of embryos transferred (2 vs. 1)	2.578	(1.457–4.456)	0.001	2.708	(1.510–4.856)	0.001
P4 < 10 ng/ml + DYD	1.304	(0.652–2.607)	0.453	1.163	(0.560–2.418)	0.685

**Abbreviations:** AMH, anti-Müllerian hormone; CI, confidence interval; DYD, dydrogesterone; OR, odds ratio.

## Discussion

There seems to be a relationship between circulating P4 in the peri-implantation period and the transfer outcome. Accumulating evidence suggest a negative relation between lower levels of P4 and treatment outcome. 13 14 18 19 23 24 25 26 27 28 29 We sought to determine whether a rescue strategy using DYD starting immediately after a blastocyst transfer could improve the outcome of cycles with low P4 values on the day of a blastocyst transfer. Our results seem to demonstrate that women with lower levels of plasmatic P4 on the day of transfer of a blastocyst can indeed be rescued with the DYD.

A similar rescue strategy concept had already been presented by Cédrin-Durnerin et al. 23 using vaginal P4 (cut-off of 10 ng/mL), and by Brady et al. 20 with intramuscular P4 (cut-off of 20 ng/mL). In both cases, the measurement of serum P4 was performed on the day of the transfer, and the patient was supplemented with an increase in the dose of the medication already on course, if the values were below the cut-offs established in the respective cohort. Both studies failed to show any improvement in terms of pregnancy outcomes. Recently, a retrospective study assessing 599 cycles concluded that hCG positive and clinical pregnancy rates were comparable between DYD three times a day and micronized vaginal P4, 30 but no sub-analysis regarding the serum progesterone values was performed. Conversely, we opted to use DYD after taking into consideration several clinical trial that evaluated the efficacy of its oral form in luteal phase support, both in fresh and frozen transfers. 11 16 17 26 31 32 33 34 35 36 37

The cut-off used for DYD administration was set according to a previous evaluation of our own population which corresponds to 11.5% of the pool of the cycles. 28

However, when compared to other series, 18 19 the P4 distribution in our population seems to be much higher. For this reason, we decided to assess what occurred in the 20% lower cycles with P4 above 10 ng/mL and we concluded that this group presented a statistically significant lower rate of hCG positive pregnancies. For this reason, we believe that center-specific rescue strategy, based on a percentile distribution of serum P4, might be better than an arbitrary threshold derived from other publications. According to our data, we believe that FET cycles in the 30% lower level of P4 might benefit from this strategy.

Two recent publications support our conclusions. Specifically, Álvarez et al. 38 reported that the addition of progesterone injections whenever serum P4 levels (measured the day prior to an euploid blastocyst transfer) were < 10.6 ng/mL was an effective rescue strategy in terms of clinical, ongoing pregnancy rate and live birth rates. Furthermore, Vuong et al. 39 also recently reported on a prospective open-label nonrandomized study with two groups of patients. One group performed luteal support with vaginal micronized progesterone 400 mg twice daily plus oral DYD 10 mg twice daily (732 patients), while the other had only vaginal micronized progesterone 400 mg twice daily (632 patients). The researchers concluded that live birth rates were statistically significantly better in the vaginal progesterone plus DYD group (46.3 vs. 41.3%).

Concerning safety issues on the use of DYD in early pregnancy, the literature is sparse. A review of 28 cases of potential links between maternal DYD use during pregnancy and congenital birth defects was reported, 40 but no conclusions could be drawn. Although Mahmoud et al. 41 found a positive association between its use in embryonic phases and congenital heart disease in the fetus, in the LOTUS I trial 16 the rates of adverse effects associated with treatment, including congenital, familial, and genetic effects were similar between the DYD and micronized vaginal P4 group (1.0% DYD vs. 1.2% MVP). A recent systematic review on the assessment of the evidence on the efficacy and safety of oral DYD versus micronized vaginal P4 for luteal phase support reported an overall similar incidence of congenital, familial, and genetic disorders between both groups. 42

The strengths of our study include the fact that that data come from a single center, during a short period of time (16 months) and that all the patients went through the same protocol. Furthermore, all serum P4 assessments were performed in the same laboratory during the morning. Conversely, the interpretation of our results may be limited by the fact that this is still a retrospective study presenting some heterogeneity between groups in terms of patient and cycle characteristics. According to our results transferring 1 or 2 embryos and transferring a day 5 or day 6 embryo might be related to the outcome. However, we tried to control for these variables, with both groups having a quite similar distribution of both these variables ( Table 1 ).

## Conclusion

In conclusion, DYD 10 mg three times a day, administered in women who perform a FET with P4 serum levels below 10 ng/mL, allows this group to have pregnancy rates beyond 12 weeks at least as good as those with serum levels above 10 ng/mL.
